# Origin, Branching, and Communications of the Intercostobrachial Nerve: a Meta-Analysis with Implications for Mastectomy and Axillary Lymph Node Dissection in Breast Cancer

**DOI:** 10.7759/cureus.1101

**Published:** 2017-03-17

**Authors:** Brandon Michael Henry, Matthew J Graves, Jakub R Pękala, Beatrice Sanna, Wan Chin Hsieh, R. Shane Tubbs, Jerzy A Walocha, Krzysztof A Tomaszewski

**Affiliations:** 1 Department of Anatomy, Jagiellonian University Medical College, Krakow, Poland; 2 Faculty of Medicine & Surgery, University of Cagliari; 3 First Faculty of Medicine, Charles University; 4 Neurosurgery, Seattle Science Foundation

**Keywords:** axillary dissection, breast cancer, intercostobrachial nerve, mastectomy, neuropathy, oncological surgery

## Abstract

The intercostobrachial nerve (ICBN), which usually originates from the lateral cutaneous branch of the second intercostal nerve, innervates areas of the axilla, lateral chest, and medial arm. It is at risk for injury during operative procedures that are often used in the management of breast cancer and such injury has been associated with postoperative sensory loss and neuropathic pain, decreasing the quality of life.

PubMed, Excerpta Medica Database (EMBASE), ScienceDirect, Google Scholar, China National Knowledge Infrastructure (CNKI), Scientific Electronic Library Online (SciELO), Biosciences Information Service (BIOSIS), and Web of Science were searched comprehensively. Data concerning the prevalence, branching, origin and communications of the ICBN were extracted and pooled into a meta-analysis.

A total of 16 studies (1,567 axillas) reported data indicating that the ICBN was present in 98.4% of person. It most often (90.6%) originated from fibers at the T2 spinal level and commonly coursed in two branching patterns: as a single trunk in 47.0% of cases and as a bifurcating pattern in 42.2%. In the latter cases, the bifurcation was usually unequal (63.4%). Additionally, the ICBN presented with anastomosing communication to the brachial plexus in 41.3% of cases.

The ICBN is a prevalent and variable structure at significant risk for injury during operative procedures of the axilla. In view of the postoperative pain and paresthesia experienced by patients following injury, surgeons need to exercise caution and aim to preserve the ICBN when possible. Ultimately, careful dissection and knowledge of ICBN anatomy could allow postoperative complications to be reduced and patient's quality of life increased.

## Introduction and background

The intercostobrachial nerve (ICBN) is a nerve classically originating from the lateral cutaneous branch of the second intercostal nerve [1]. The ICBN functions to innervate portions of the axilla, tail of the breast, lateral chest wall and medial side of the arm [2-3]. The ICBN, in its historically portrayed course, exits the second intercostal space and traverses the axilla to terminally branch in the region of the medial arm [1]. The proximity and course of the ICBN in relation to the axilla poses a danger of iatrogenic injury resulting from common procedures such as axillary lymph node dissection (ALND), sentinel lymph node biopsy (SLNB) and mastectomy [2,4].

Breast cancer is the most common malignancy in women worldwide, affecting nearly one in eight in both the United States and Europe [1, 5-6]. Procedures such as ALND, SLNB, and/or mastectomy are utilized in a significant portion of breast cancer cases for purposes ranging from diagnosis, staging, to resection [1, 7-8]. The ICBN is the most commonly injured nerve during mastectomy and is believed to be implicated in both persistent pain after breast cancer treatment (PPBCT) and permanent loss of sensory function in the region supplied [1-2,9].

It has been estimated that between 10-60% of breast cancer survivors who have undergone operative procedures, experience PPBCT, and it is believed that this predominantly neuropathic pain contributes significantly to a reduced quality of life following breast cancer treatment [1, 9-10]. Aside from dysesthesia, paresthesia of the ICBN, sensory distribution is also very common [11]. It has been shown that preservation of the ICBN provides for a clear reduction in post-procedural paresthesia and improves the quality of life [1, 11]. Surgeons should exercise precision during dissections of the axillary region as early branching and anastomosing fibers between the ICBN and brachial plexus can easily be overlooked [1, 12-13].

ICBN anatomy has been studied in detail in the past two decades, yet on a very limited scale. The ICBN’s structure is highly variable in nature with numerous origins, branching patterns and communicating branches [1, 4-13-17]. Determining the true prevalence and nature of the variant patterns of ICBN branching and origin will serve surgeons to better preserve these neural structures. A surgeon’s knowledge of these structures is vital to their safeguarding during operative breast cancer management. The clinical importance of the ICBN necessitates further investigations in the future, because despite the development of detailed anatomical investigations that are becoming more widespread, the ICBN remains an overlooked structure.

The aim of this study was to utilize all available data to date to provide a comprehensive evidence-based appraisal of the prevalence and varying patterns of ICBN origin and branching. It is our intention, that this information be utilized to help decrease the morbidity and risk of postoperative complications of breast cancer treatment.

## Review

### Methods

Search Strategy

Identification of articles for inclusion in the meta-analysis was performed with searches through December 2016 in the following databases: PubMed, EMBASE, ScienceDirect, Google Scholar, China National Knowledge Infrastructure (CNKI), SciELO, BIOSIS, and Web of Science. The search strategy executed in PubMed is presented as follows:

(Intercostobrachial nerve[title/abstract]) or nervus intercostobrachialis[title/abstract]

No date or language restrictions were applied. Identification of additional studies eligible for the meta-analysis was performed through searching the references of all included articles. Throughout the meta-analysis, adherence to the preferred reporting items for systematic reviews and meta-analyses (PRISMA) guidelines were exercised (See Appendix - Table 5) [18].

Eligibility Assessment

Eligibility for inclusion in the meta-analysis was assessed by two independent reviewers. All intraoperative or cadaveric studies that reported extractable prevalence data of ICBN origin or branching were included. The exclusion criteria included: case reports, case series, letters to the edito or conference abstracts. All studies published in languages not fluently spoken by any of the authors were translated by medical professionals fluent in both English and the language of the original manuscript. Disagreements between reviewers arising during the eligibility assessment process were resolved by discussion and consensus.

Data Extraction

Data from included studies was independently extracted by three reviewers. Extracted data included year, country, sample size (number of patients and number of axillas), prevalence of ICBN, ICBN origin, branching pattern of ICBN and the prevalence of communication with the brachial plexus. In the event of any discrepancies in the data, authors of the original were contacted for clarification when possible.

Statistical Analysis

The single-categorical and multi-categorical pooled prevalence estimates were calculated using MetaXL version 2.0 by EpiGear Pty Ltd. (Wilston, Queensland, Australia) [19]. A random effects model was used for all statistical analyses. Heterogeneity was assessed by both the Chi^2^ test and the I^2^ statistic. For the Chi^2^ test, a p-value of < 0.10 for Cochran’s Q served as an indicator of significant heterogeneity among the studies analyzed [20]. The results of the I^2^ statistic were interpreted as follows: zero percent to 40% might not be important; 30%- 60% could indicate moderate heterogeneity; 50% - 90% could indicate substantial heterogeneity; and 75% - 100% could represent considerable heterogeneity [20].

Subgroup analysis was performed on the basis of type of study (cadaveric vs. operative) and geographical origin of the study. Statistically significant differences between analyzed groups were determined by their confidence intervals. If the confidence intervals of any two rates overlapped, the differences were regarded as statistically insignificant [19]. Lastly, sensitivity was assessed by a separate analysis of studies with sample sizes greater than 100 to probe further for potential sources of heterogeneity.

### Results

Study Identification

**Figure 1:**
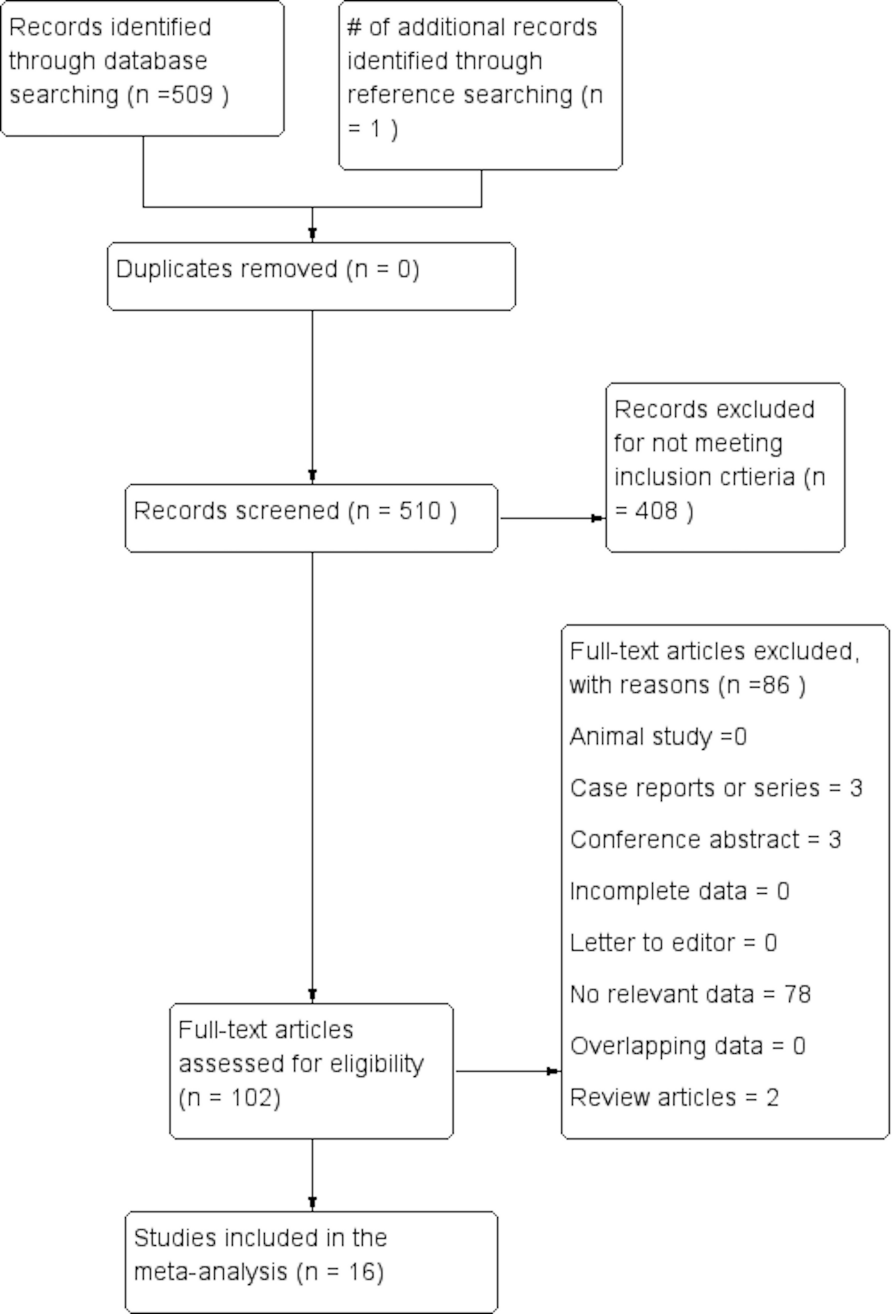
PRISMA flow chart of the study identification and inclusion in the meta-analysis

The flow of studies through the meta-analysis is presented in Figure 1. The search of the major electronic databases identified an initial 509 articles, with an additional one article identified in the search of the references of included studies. A total of 102 articles were assessed for eligibility using full-texts, of which 86 were excluded and 16 were included into the meta-analysis.

Characteristics of Included Studies

The characteristics of the studies that have been included in the meta-analysis are summarized in Table 1. Sixteen studies (1,567 axillas) [1, 3-4, 13,17, 21,28] were included in the meta-analysis. Five studies were performed on cadaveric specimens [13, 15, 22, 25, 27], 10 were performed intraoperatively [1,3-4, 14, 16- 17, 21, 24, 26, 28], and one study [23] examined both cadavers and operative subjects. The studies ranged in year from 1999 to 2016 and had a vast geographic breakdown with 10 from Asia, three from Europe, and one each from North America, South America, and Oceania.

**Table 1:** Characteristics of included studies ICBN - intercostobrachial nerve

Study	Country	Type	n = (# half-bodies studied)	# of half-bodies with ICBN (%)
O’Rourke 1999	Australia	Cadaveric	28	28 (100%)
Cunnick 2001	United Kingdom	Operative	50	45 (90%)
Wu 2001	China	Operative	204	203 (100%)
Yin 2004	China	Cadaveric	50	48 (96%)
Li 2005	China	Cadaveric & Operative	70	70 (100%)
Zhao 2005	China	Operative	151	151 (100%)
Ge 2006	China	Cadaveric	5	5 (100%)
Loukas 2006	Grenada/USA	Cadaveric	200	200 (100%)
Zhang 2006	China	Operative	216	207 (96%)
Zhao 2008	China	Cadaveric	32	31 (97%)
Verma 2009	India	Operative	69	69 (100%)
Khan 2012	United Kingdom	Operative	73	73 (100%)
Kubala 2013	Czech Republic	Operative	113	107 (95%)
Soares 2014	Brazil	Operative	100	99 (99%)
Zhu 2014	China	Operative	156	156 (100%)
Kumar 2016	India	Operative	50	50 (100%)

Prevalence of the Intercostobrachial Nerve

**Figure 2:**
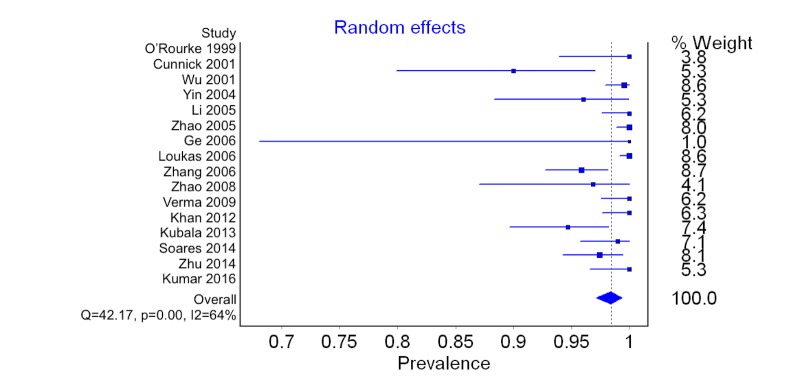
Forest plot for pooled prevalence of the intercostobrachial nerve

A total of 16 studies (1,567 axillas) reported data on the prevalence of the ICBN. The overall pooled prevalence of the ICBN in axillas was 98.4% (95%CI: 97.1-99.4) (Figure 2). No significant differences in prevalence were noted during geographic or study type subgroup analysis. Sensitivity analysis also proved to not reveal any notable differences. Data on the prevalence of the ICBN is presented in Table 2.

**Table 2:** Prevalence of the intercostobrachial nerve in human axillas

Category	# of studies (# of axillas)	Prevalence of ICBN: % (95% CI)	I^2^: % (95% CI)*
Overall	16 (1567)	98.4 (97.1-99.4)	64.4 (39.3-79.1)
Cadaveric	5 (315)	98.3 (95.3-100)	51.0 (0.0-82.0)
Operative	10 (1182)	98.3 (96.5-99.4)	70.5 (43.5-84.5)
Asia	10 (1003)	98.6 (97.2-99.6)	53.4 (4.7-77.2)
Europe	3 (236)	95.7 (88.6-100)	80.5 (38.9-93.8)
Sensitivity (n≥100)	7 (1140)	98.6 (96.7-99.7)	76.7 (51.4-88.9)

Origin of the intercostobrachial nerve

Twelve studies (922 ICBNs) reported data on the origin of the ICBN. The most common origin was from the T2 vertebral level, with 90.6% (95%CI: 83.1-98.7) of ICBNs originating at that level. A pure T2 origin was followed by T2 & T3 combined origin [3.4% (95%CI: 0.0-9.7)] and T1 & T2 combined origin [2.9% (95%CI: 0.0-8.8)]. To further clarify, combined origin was listed as such when two separate roots were observed unifying into one common ICBN. Subgroup and sensitivity analysis did not yield any significant findings or differences between groups. Data regarding the origin of the ICBN is presented in Table 3.

**Table 3:** Types of origin of the intercostobrachial nerve from the lateral cutaneous branches of the intercostal nerves (vertebral levels)

Category	# of studies (# of nerves)	T1 % (95% CI)	T1 & T2 % (95% CI)	T1, T2, & T3 % (95% CI)	T2 % (95% CI)	T2 & T3 % (95% CI)	T3 % (95% CI)	T2, T3, & T4 % (95% CI)	I^2^: % (95% CI)*
Overall	12 (922)	0.8 (0.0-4.5)	2.9 (0.0-8.8)	1.1 (0.0-5.1)	90.6 (83.1-98.7)	3.4 (0.0-9.7)	0.6 (0.0-4.0)	0.6 (0.0-4.0)	94.3 (91.8-96.1)
Cadaveric	5 (312)	0.8 (0.0-13.9)	1.4 (0.0-16.2)	0.8 (0.0-13.9)	88.3 (68.4-100)	6.4 (0.0-29.7)	0.8 (0.0-13.9)	1.4 (0.0-16.0)	95.8 (92.8-97.6)
Operative	7 (568)	1.0 0.0-3.6)	4.9 (1.2-10.6)	1.5 (0.0-4.5)	89.7 (82.6-95.4)	2.0 (0.0-6.3)	0.6 (0.0-2.8)	0.4 (0.0-2.2)	82.9 (66.1-91.3)
Asia	8 (576)	1.0 (0.0-5.3)	3.2 (0.0-9.7)	1.5 (0.0-6.3)	92.0 (87.5-100)	1.3 (0.0-5.8)	0.5 (0.0-3.7)	0.5 (0.0-3.7)	91.1 (85.0-94.8)
Europe	2 (118)	0.4 (0.0-7.4)	3.4 (0.0-16.0)	0.4 (0.0-7.4)	90.5 (71.8-100)	3.2 (0.0-15.5)	1.7 (0.0-11.8)	0.4 (0.0-7.4)	86.7 (47.8-96.6)
Sensitivity (n≥100)	3 (503)	1.4 (0.0-15.6)	5.4 (0.0-26.1)	2.8 (0.0-19.8)	76.8 (38.9-98.8)	12.9 (0.0-39.8)	0.6 (0.0-12.2)	0.2 (0.0-9.3)	98.2 (96.8-99.0)

Branching of the Intercostobrachial Nerve

**Figure 3:**
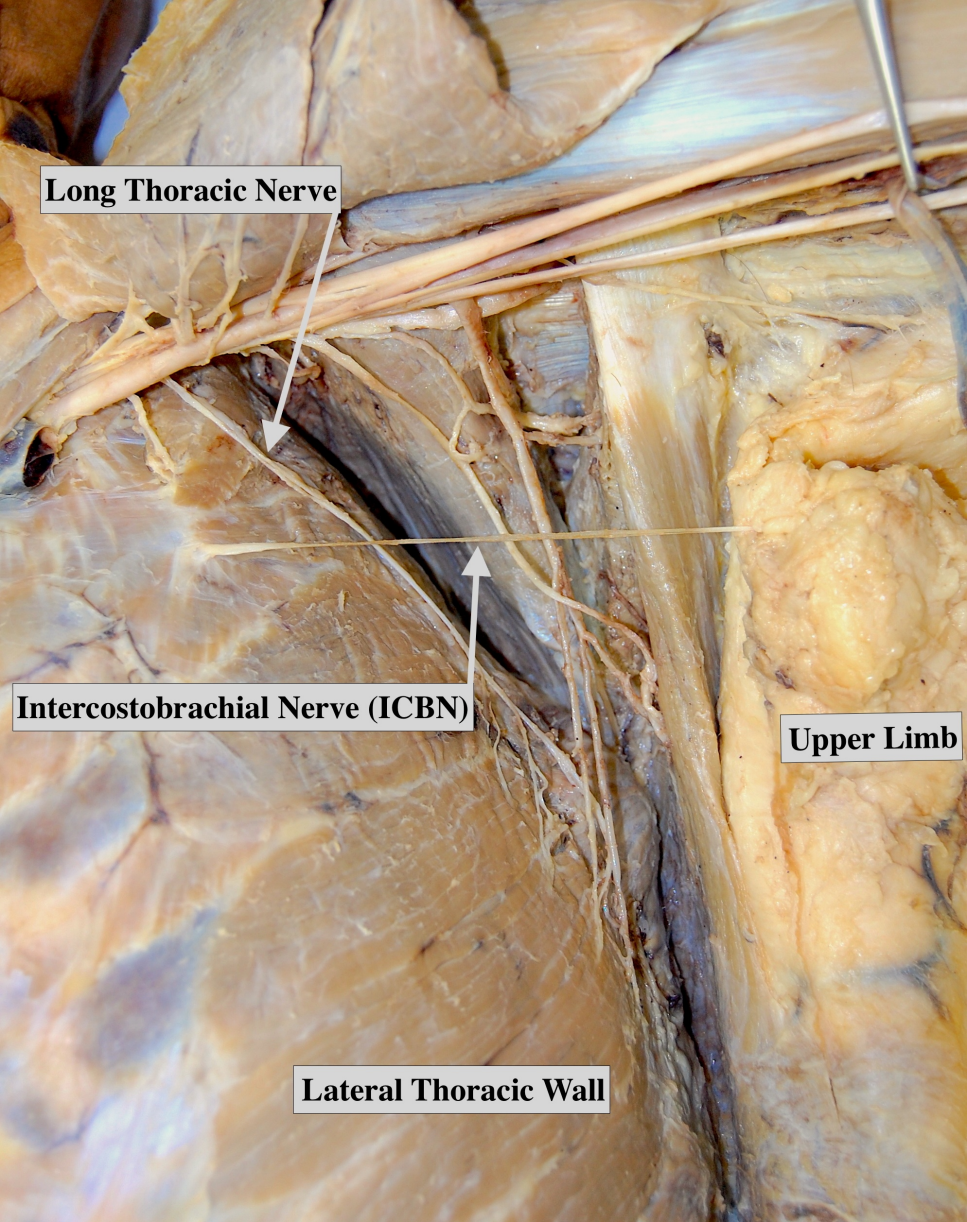
Cadaver displaying a single trunk intercostobrachial nerve

**Figure 4:**
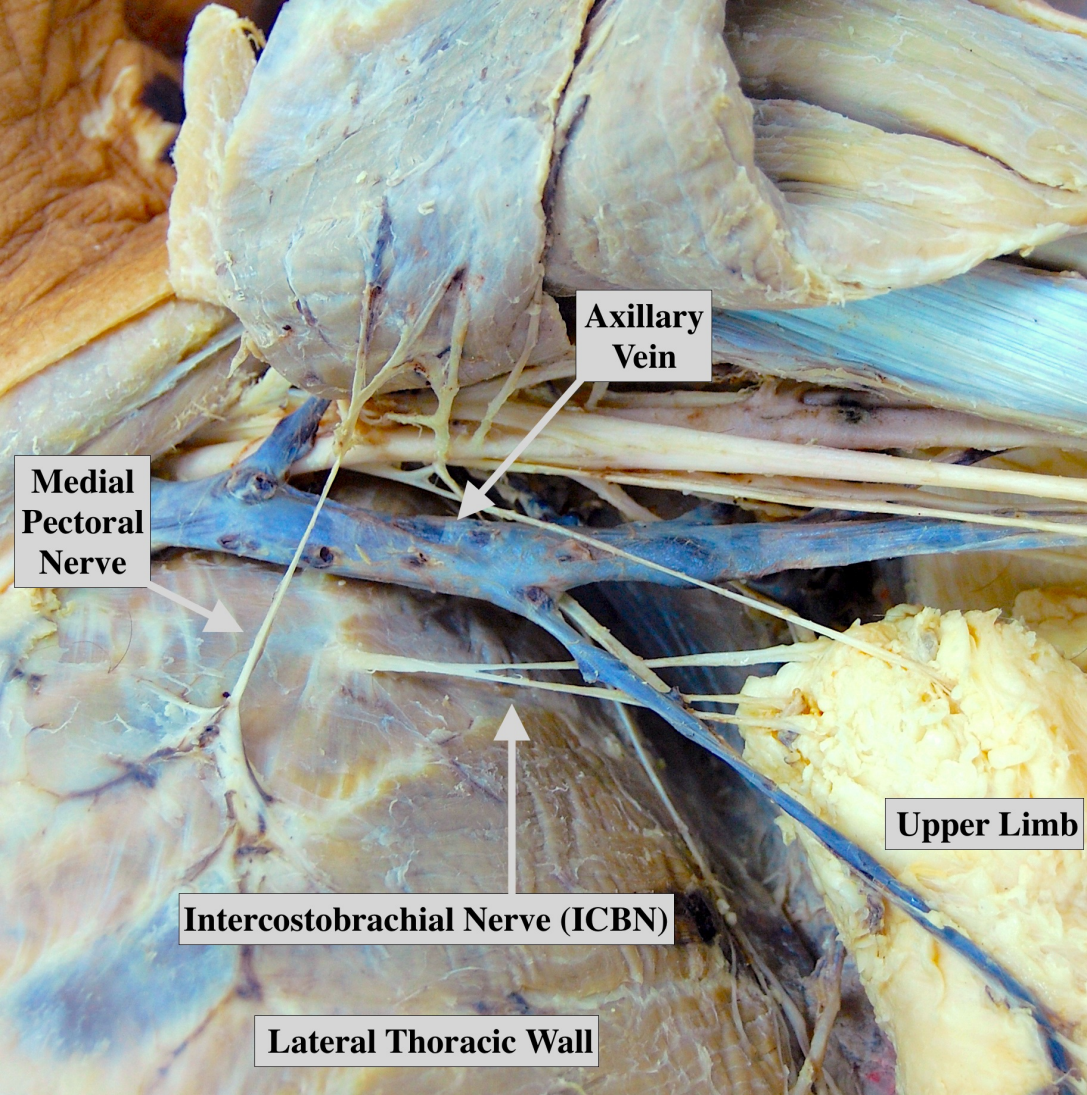
Cadaver displaying a bifurcating intercostobrachial nerve

A total of 12 studies (1,234 ICBNs) reported data on the branching pattern of the ICBN. Detailed information on the ICBN branching data is presented in Table 4. The ICBN most commonly existed as a single trunk [47.0% (95%CI: 23.8-67.9)] (Figure 3) followed closely by a bifurcating pattern [42.2% (95%CI: 19.8-63.4)] (Figure 4). Subgroup analysis revealed that the presence of a bifurcating ICBN was more frequently seen in cadaveric studies versus intraoperative studies [65.3% (95%CI: 13.6-100) vs. 30.2% (95%CI: 6.3-57.6)], albeit not significantly. Further subgroup and sensitivity analysis did not reveal any significant findings.

**Table 4:** Branching patterns of the intercostobrachial nerve

Category	# of studies (# of nerves)	Single Trunk % (95% CI)	Unification of two branches into Single Trunk % (95% CI)	Unification of two branches into Single Trunk with Re-Branching % (95% CI)	Total Bifurcation % (95% CI)	Multiple Branches % (95% CI)	I^2^: % (95% CI)*
Overall	12 (1234)	47.0 (23.8-67.9)	2.7 (0.0-12.0)	0.6 (0.0-6.5)	42.2 (19.8-63.4)	7.5 (0.0-20.8)	98.2 (97.7-98.6)
Cadaveric	4 (284)	23.9 (0.0-75.9)	2.9 (0.0-35.2)	0.9 (0.0-26.4)	65.3 (13.6-100)	7.0 (0.0-46.9)	97.6 (96.0-98.6)
Operative	8 (950)	59.2 (27.4-83.1)	2.5 (0.0-15.0)	0.5 (0.0-8.5)	30.2 (6.3-57.6)	7.6 (0.0-25.3)	98.6 (98.1-98.9)
Asia	9 (916)	39.9 (11.8-69.3)	1.2 (0.0-12.1)	0.5 (0.0-9.3)	50.5 (19.7-78.5)	7.9 (0.0-27.2)	98.6 (98.1-98.9)
Europe	2 (118)	60.3 (25.8-89.6)	9.5 (0.0-33.0)	1.7 (0.0-15.6)	23.3 (1.0-56.5)	5.3 (0.0-24.8)	91.4 (70.0-97.5)
Sensitivity (n ≥100)	5 (913)	61.8 (19.0-93.8)	0.9 (0.0-16.3)	0.1 (0.0-11.4)	29.1 (0.0-65.6)	7.9 (0.0-34.7)	99.2 (98.9-99.4)

Bifurcation Type

Eight studies (381 bifurcating ICBNs) reported data on the specific pattern of bifurcation. It was revealed that 36.6% (95%CI: 25.2-48.7) of ICBNs bifurcate into equal branches, that is specifically branches of equal size. Unequal bifurcation was observed in 63.4% (95%CI: 51.3-74.8) of cases and was denoted when branches following a bifurcation were not of equal size [I^2^=79.0% (95%CI: 59.0-89.2), Cochran’s Q, p <0.001].

Communication with the Brachial Plexus

Four studies (479 ICBNs) included information of neural anastomoses with the brachial plexus (medial cord or medial brachial cutaneous nerve of the arm]. It was noted that 41.3% (95%CI: 6.8-80.6) of ICBNs had these communicating branches [I^2^=98.3% (95%CI: 97.3-98.9), Cochran’s Q, p < 0.001].

### Discussion

The ICBN is a nerve of significant importance in surgical procedures involving the axillary region. Damage to the ICBN is associated with significant morbidity and is believed to be involved with postoperative pain and sensory loss [1, 9-10].

Our analysis demonstrated the overall prevalence of the ICBN to be 98.4% and that it most often exists as a single trunk (47.0%) originating from the T2 vertebral level (90.6%). Subgroup analysis revealed little variation in the results aside from that of ICBN branching. Branching subgroups differed largely based on the modality of the study (cadaveric vs. operative). When examining the statistics for bifurcation alone, it is notable that ICBNs examined in cadavers (65.3%) had more than twice the rate of bifurcation than those assessed intraoperatively (30.2%). We posit two concepts from these differences. The first being that due to the limited field of view intraoperatively, as well as the inability to freely dissect tissue without consequences, may limit the branches which are identified in-vivo. It should be noted that these differences may help explain why nerves which are successfully identified and protected can still present with postoperative complications [1]. It was noted in Kumar (2016) that six months following the procedure, 20% of patients who had successful ICBN preservation still experienced numbness and paresthesia [1]. However, in addition to a bifurcating ICBN, this may also be due to stretching of the nerve by retractors or other intraoperative stress placed on the nerve fibers. The secondary factor contributing to the cadaveric-intraoperative discord may be that nerves that do bifurcate, have a propensity to do so in an unequal fashion (63.4% unequal versus 36.6% equal bifurcation). As such, surgeons may easily identify the larger trunk but neglect to identify the smaller branch of the nerve, placing it at a higher risk of operative injury.

Preservation of the ICBN is very important and should be factored into operative plans. There are cases where ICBN conservancy will not be possible, but in those where it is possible, it was associated with an insignificant increase in operative time of approximately five minutes [1].

The neural fibers travelling within the ICBN appear to vary, as there are cases of known ICBN loss where patients have no postoperative neural deficits [1]. As it is difficult to determine preoperatively the role of the ICBN in a patient, a vital sensory function of the nerve should be assumed. It was also discovered in our analysis that 41.3% of ICBNs contributed anastomosing branches to the brachial plexus, namely the medial cord or medial brachial cutaneous nerve. This lends some explanation to the unpredictability of deficits that can present in patients [13]. Losses in sensation or pain have been noted in locations ranging from the medial side of the proximal arm, axillary region, to the anterior chest wall [1, 17].

Various techniques of dissection have been proposed throughout the history of ICBN examination. Some techniques advocate that a superficial blunt dissection should be conducted from the site of axillary incision towards the lateral edge of the pectoralis muscle [12]. The ICBN can be identified consistently in an anterior position during exposure of the long thoracic and thoracodorsal nerves [13, 29-30]. Extra care should be particularly exercised in the area of the second intercostal space where ICBN origin is most likely (90.6%). Surgeons should however, consider than many ICBNs do receive fibers from either the first or third intercostal nerves as well. Some also note that dissection could propagate inferiorly from an axillary incision, traveling from the axillary vessels, layer-by-layer until positive nerve identification [4]. It is also noted that in the area of the inferior margin of the pectoralis minor muscle and dissection deep to the pectoralis minor, should be avoided if possible due to risks of ICBN injury [31].

Operative procedures of the axilla are often associated with varying stages of breast cancer management. The ICBN can be damaged from any number of reasons ranging from traction to transection [32]. Additionally, damaged nerves may also develop neuromas that can further complicate the clinical picture [1, 32]. Surgeons in some cases must make decisions that have consequences in order to get the proper margins in oncologic surgery. It should, however, be standard to exercise protective practices towards the ICBN when possible. ICBN neuralgia and post-mastectomy pain syndrome can be successfully managed with loco-regional anesthetic techniques, however, overall reduction in incidence should be the primary goal [4, 32].

Our meta-analysis was limited by a number of factors involved in inconsistent reporting and small sample sizes. Some studies presented origins from the first intercostal nerve or third intercostal nerve as separate data. It was unclear to whether these origins were duplications or simply contributing fibers and thus had to be excluded from analyses where this information could have skewed results. Additionally, high heterogeneity persisted despite our subgroup analyses, thus we postulate this may have resulted from the intrinsically variable nature of the ICBN. This high level of heterogeneity was sustained in defiance of the fact that pooled prevalences were largely consistent between groups, pointing further towards the notion that there is indeed high anatomic variability. There was an overall lack of reporting by studies on factors such as gender and laterality, which could have provided for additional subgroup analyses. The overwhelming majority of studies were performed on women, however, Loukas (2006) noted no differences between genders [15]. Limited data from regions such as Africa, North America, Oceania and South America precluded analyses of other regions aside from Europe and Asia. Detailed data was absent from many studies regarding branching site or details on the symmetry of the post-division branches.

Future studies need to be performed to further elucidate the behavior of the terminal branching of the ICBN as well as its anastomotic behavior with the brachial plexus. There is also the possibility to investigate the use of landmarks or relationships with adjacent structures as a means for intraoperative nerve identification. Structures such as the lateral thoracic vein discussed in O’Rourke (1999) could be a potential candidate [13]. Finally, histologic studies should be performed to examine for possibilities of microscopic branching and further identify the types of neural fibers running within the nerve. Investigating this may help explain cases of postoperative neuropathy where nerves were successfully spared intraoperatively. Supplementation with future research will provide valuable information for surgeons and ideally result in more positive outcomes for patients undergoing procedures in the region.

## Conclusions

The ICBN is a prevalent anatomical structure with significant variability in origin and branching pattern. It is at considerable risk during operative procedures in the axilla, especially those involved in breast cancer management. Injury to ICBN fibers has been associated with post-procedural pain, paresthesia, and ultimately reduced the quality of life. Surgeons need to exercise caution and should adopt measures to protect the ICBN, its branches, and anastomosing fibers if possible. The information presented in this study, accompanied by careful surgical practice, could help to reduce significant complications during mastectomy and axillary dissection.

## Appendices

**Table 5:** PRISMA 2009 checklist Completed checklist for the preferred reporting items for systematic reviews and meta-analyses (PRISMA) 2009 guidelines

Section/topic	#	Checklist item	Reported on page #
TITLE			
Title	1	Identify the report as a systematic review, meta-analysis, or both.	1
ABSTRACT			
Structured summary	2	Provide a structured summary including, as applicable: background; objectives; data sources; study eligibility criteria, participants, and interventions; study appraisal and synthesis methods; results; limitations; conclusions and implications of key findings; systematic review registration number.	2
INTRODUCTION			
Rationale	3	Describe the rationale for the review in the context of what is already known.	4-5
Objectives	4	Provide an explicit statement of questions being addressed with reference to participants, interventions, comparisons, outcomes, and study design (PICOS).	4-5
METHODS			
Protocol and registration	5	Indicate if a review protocol exists, if and where it can be accessed (e.g., Web address), and, if available, provide registration information including registration number.	6
Eligibility criteria	6	Specify study characteristics (e.g., PICOS, length of follow-up) and report characteristics (e.g., years considered, language, publication status) used as criteria for eligibility, giving rationale.	6
Information sources	7	Describe all information sources (e.g., databases with dates of coverage, contact with study authors to identify additional studies) in the search and date last searched.	6
Search	8	Present full electronic search strategy for at least one database, including any limits used, such that it could be repeated.	6
Study selection	9	State the process for selecting studies (i.e., screening, eligibility, included in systematic review, and, if applicable, included in the meta-analysis).	6
Data collection process	10	Describe method of data extraction from reports (e.g., piloted forms, independently, in duplicate) and any processes for obtaining and confirming data from investigators.	6-7
Data items	11	List and define all variables for which data were sought (e.g., PICOS, funding sources) and any assumptions and simplifications made.	6-7
Risk of bias in individual studies	12	Describe methods used for assessing risk of bias of individual studies (including specification of whether this was done at the study or outcome level), and how this information is to be used in any data synthesis.	N/A
Summary measures	13	State the principal summary measures (e.g., risk ratio, difference in means).	6-7
Synthesis of results	14	Describe the methods of handling data and combining results of studies, if done, including measures of consistency (e.g., I^2^) for each meta-analysis.	7

Section/topic	#	Checklist item	Reported on page #
Risk of bias across studies	15	Specify any assessment of risk of bias that may affect the cumulative evidence (e.g., publication bias, selective reporting within studies).	N/A
Additional analyses	16	Describe methods of additional analyses (e.g., sensitivity or subgroup analyses, meta-regression), if done, indicating which were pre-specified.	7
RESULTS			
Study selection	17	Give numbers of studies screened, assessed for eligibility, and included in the review, with reasons for exclusions at each stage, ideally with a flow diagram.	8
Study characteristics	18	For each study, present characteristics for which data were extracted (e.g., study size, PICOS, follow-up period) and provide the citations.	8
Risk of bias within studies	19	Present data on risk of bias of each study and, if available, any outcome level assessment (see item 12).	N/A
Results of individual studies	20	For all outcomes considered (benefits or harms), present, for each study: (a) simple summary data for each intervention group (b) effect estimates and confidence intervals, ideally with a forest plot.	8-9
Synthesis of results	21	Present results of each meta-analysis done, including confidence intervals and measures of consistency.	8-9
Risk of bias across studies	22	Present results of any assessment of risk of bias across studies (see Item 15).	N/A
Additional analysis	23	Give results of additional analyses, if done (e.g., sensitivity or subgroup analyses, meta-regression [see Item 16]).	8-9
DISCUSSION			
Summary of evidence	24	Summarize the main findings including the strength of evidence for each main outcome; consider their relevance to key groups (e.g., healthcare providers, users, and policy makers).	10-13
Limitations	25	Discuss limitations at study and outcome level (e.g., risk of bias), and at review-level (e.g., incomplete retrieval of identified research, reporting bias).	12-13
Conclusions	26	Provide a general interpretation of the results in the context of other evidence, and implications for future research.	13
FUNDING			
Funding	27	Describe sources of funding for the systematic review and other support (e.g., supply of data); role of funders for the systematic review.	13
